# Transient Heat Conduction in the Orthotropic Model with Rectangular Heat Source

**DOI:** 10.3390/mi13081324

**Published:** 2022-08-16

**Authors:** Zeqing He, Yingli Shi, Yuqing Shen, Zhigang Shen, Taihua Zhang, Zhao Zhao

**Affiliations:** 1 Aerospace Information Research Institute, Chinese Academy of Sciences, Beijing 100094, China; 2 School of Materials and Energy, University of Electronic Science and Technology of China (USETC), Chengdu 610054, China; 3 Institute of Solid Mechanics, Beihang University (BUAA), Beijing 100191, China; 4 Beijing Key Laboratory for Powder Technology Research and Development, Beihang University (BUAA), Beijing 100191, China; 5 China Special Equipment Inspection and Research Institute, Beijing 100029, China

**Keywords:** orthotropic substrate, transient heat conduction, stretchable rectangular heater, uniform temperature distribution

## Abstract

Epidermal electronic systems (EESs) are a representative achievement for utilizing the full advantages of ultra-thin, stretchable and conformal attachment of flexible electronics, and are extremely suitable for integration with human physiological systems, especially in medical hyperthermia. The stretchable heater with stable electrical characteristics and a uniform temperature field is an irreplaceable core component. The inorganic stretchable heater has the advantage of maintaining stable electrical characteristics under tensile deformation. However, the space between the patterned electrodes that provides tensile properties causes uneven distribution of the temperature field. Aiming at improving the temperature distribution uniformity of stretchable thermotherapy electrodes, an orthotropic heat transfer substrate for stretchable heaters is proposed in this paper. An analytical model for transient heat conduction of stretchable rectangular heaters based on orthotropic transfer characteristics is established, which is validated by finite element analysis (FEA). The homogenization effect of orthotropic heat transfer characteristics on temperature distribution and its evolutionary relationship with time are investigated based on this model. This study will provide beneficial help for the temperature distribution homogenization design of stretchable heaters and the exploration of its transient heat transfer mechanism.

## 1. Introduction

As a representative of flexible electronics [1,2,3,4], the epidermal electronic system (EES) [5,6] is a remarkable achievement that utilizes the full advantages of ultra-thin, stretchable and conformal attachment, and is extremely suitable for integration with human physiological systems. Among the many EESs with various functions [7,8], the structure and function of the flexible epidermal heater are relatively simple, but it has an indispensable wide range of applications [9,10]. Especially in medical epidermal hyperthermia [11], it plays an irreplaceable role, for example, hyperthermia accelerates wound healing [12,13], subcutaneous tissue tumor treatment [14], heat control drug release [15], etc.

Flexible epidermal heaters have two flexibility strategies, i.e., material flexibility innovation and structure flexibility innovation, which are utilized in most flexible electronics and systems [16,17]. The device fabrication strategy of flexible material, namely organic flexible electronics, mainly includes the highly elastic polymer materials filled with high-conductivity nanowires or nanoparticles [18,19], stretchable organic polymers with high conductive properties [20,21], two-dimensional high-conductivity materials [22], etc. Benefitting from the intrinsic flexibility of the material, devices based on this fabrication strategy have excellent stretchability properties. However, their obvious limitation is that the tensile deformation will lead to the slippage of the fibers and micro-nano components in the polymer matrix materials, which will make the electrical resistance vary dramatically under stretching conditions. This leads to serious deterioration of the electrical properties of the devices with flexible deformation. As for the flexible epidermal heater, the performance degrading will lead to the mismatch between the temperature distribution and the design value. Inorganic EESs, based on the fabrication strategy of flexible packaging of patterned electronics, can transform large structural deformation into small strain through thinned rigid material and malleable structural design, thus significantly improving the stretchability of the devices and simultaneously providing excellent electrical properties of metal-based or semiconductor-based inorganic electronics [23,24,25]. The electrical performance of flexible epidermal heaters based on this approach will not deteriorate or drift significantly with the flexible deformation. In order to ensure extensibility performance, there is a certain spacing between the patterned electrodes of the flexible epidermal heater [26,27] which will bring a certain inhomogeneity to the temperature distribution of the heater. The temperature distribution uniformity is an important index to measure the function of hyperthermia heaters. Inhomogeneous temperature fields will make it difficult to render an effective hyperthermia result. Areas that are too hot may cause thermal damage to the epidermal tissue, while areas with low temperature may not be effective as a hyperthermia treatment [26].

To address this problem, using the materials with orthotropic heat conduction characteristics as the flexible substrate or encapsulation of the stretchable heater can significantly improve the uniformity of temperature distribution [28,29,30]. Li et al. proposed the thermal management design of inorganic light-emitting diodes (μ-ILEDs) with orthotropic substrate and its integration with skin, which demonstrated excellent thermal management ability verified by theoretical analysis [29,30]. Shi et al. proposed a thermal protection substrate for wearable electronics based on orthotropic characteristics and phase change temperature manipulation, which can reduce the peak temperature by more than 85% compared with the conventional substrate [31]. The orthotropic thermal meta-material formed by the materials with significant thermal conductivity differences by the way of alternately stacking arrangement has a splendid effect on heat flux manipulation [32]. The orthotropic substrate with in-plane thermal conductivity larger than off-plane thermal conductivity can better manipulate the heat flux uniformly diffused in all directions to the in-plane direction, so as to realize the temperature distribution on demand. It is a solution with low micro-nano fabrication process requirements and terrific thermal management effect, which has been utilized in the temperature distribution regulation of the flexible electronics heat transfer system [33]. For the heat conduction system of flexible epidermal heaters, the establishment of the corresponding analytical heat conduction model is a convincing and systematic approach to investigate the thermal manipulation effect [34,35,36]. In our pre-sequence work, the steady-state heat conduction model of stretchable thermal heaters is established and the factors affecting the temperature distribution are systematically investigated [37]. However, for the heating system composed of flexible materials with low thermal conductivity, the transient evolution model of temperature distribution variation with time makes more sense to clarify the thermal transfer process.

Here, an analytical model is established for the orthotropic transient heat conduction process of stretchable rectangular heat sources in order to investigate the heat flux manipulation mechanism and the temperature distribution homogenization effect with time variation. The analytical model of the transient temperature distribution is deduced in Section 2, based on Fourier heat conduction model and linear superposition principle. The corresponding finite element analysis (FEA) verification is carried out in Section 3. The parameters that affect the instantaneous temperature distribution uniformity are systematically investigated in this section. The conclusion and discussion are present in Section 4 of the article.

## 2. Analytical Modelling

**Figure 1:**
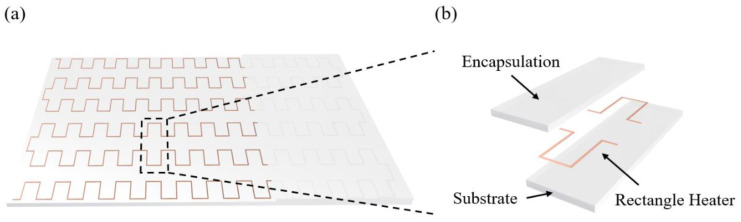
(**a**) Schematic diagram of the stretchable rectangular heater embedded in the hyper-elastic materials. (**b**) Explosion diagram of a single period heater with encapsulation and substrate.

The schematic diagram of the stretchable rectangular heater embedded in the hyper-elastic materials is shown as Figure 1a. Considering the periodic structure of the heater, the temperature distribution of the whole structure can be obtained by linear array of that of the single repeatable unit heater with encapsulation and substrate, as shown in Figure 1b.

**Figure 2:**
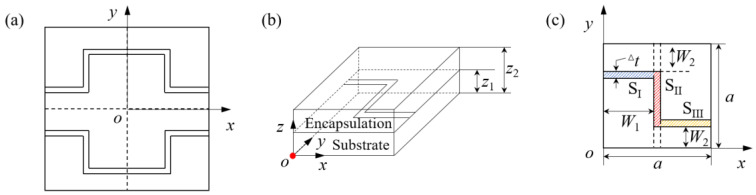
(**a**) A single period schematic diagram of the stretchable rectangular heater. A quarter schematic diagram of the heater (**b**) with encapsulation and substrate and (**c**) structural parameters.

In the analytical model, the planar heat source is used to model the stretchable heater, because the cross-sectional thickness of the heater is much smaller than the cross-sectional width and the length of the heater in the in-plane direction, as shown in Figure 2. The coordinate origin of the analytical model is set at the geometric center of the single unit, as shown in Figure 2a. Considering the geometric symmetry of the unit, a quarter of the heat conduction model was selected for investigation, as shown in Figure 2b,c.

The three-dimensional transient heat conduction in a quarter period of the stretchable rectangular heater can be expressed as

(1) $$\left\{ \begin{array}{ll} k_{x1}\frac{\partial^{2}T}{\partial x^{2}}+k_{y1}\frac{\partial^{2}T}{\partial y^{2}}+k_{z1}\frac{\partial^{2}T}{\partial z^{2}}=\rho_{1}C_{p1}\frac{\partial T}{\partial t}, & 0<z<z_{1},\;0<x<a,\;0<y<a,\\ k_{x2}\frac{\partial^{2}T}{\partial x^{2}}+k_{y2}\frac{\partial^{2}T}{\partial y^{2}}+k_{z2}\frac{\partial^{2}T}{\partial z^{2}}+Q=\rho_{2}C_{p2}\frac{\partial T}{\partial t}, & z_{1}<z<z_{2},\;0<x<a,\;0<y<a. \end{array}\right.,$$

where the heat flux density *Q* can be expressed by a series of independent variables, it can be obtained as

(2) $$Q=Q_{0}Q_{1}(x,y)Q_{2}(z)Q_{3}(t),$$

where the *Q*_0_ is the surface heat flux density, *Q*_2_(*z*) = *δ*(*z* − *z*_1_) with δ denoting the Dirac function, *Q*_3_ (*t*) represents the mode of the electronic. *Q*_1_(*x*, *y*) illustrates the effecting range of the stretchable rectangular heater which can be divided into three parts with S_I_, S_II_ and S_III_:

(3) $$Q_{1}(x,y)=\left\{ \begin{array}{l} 1,\;(x,y)\in (S_{I}\cup S_{\mathrm{II}}\cup S_{\mathrm{III}}),\\ 0,\;(x,y)\notin ({\mathrm{>S}}_{I}\cup {\mathrm{>S}}_{\mathrm{II}}\cup {\mathrm{>S}}_{\mathrm{III}}). \end{array}\right.$$

The natural convection boundary conditions are applied into the top and bottom surface and the four sides are set as adiabatic boundary condition, which yields

(4) $$\left\{ \begin{array}{l} k_{z1}{\frac{\partial T_{1}}{\partial z}|}_{z=0}=h_{1}T_{1},\\ -k_{z2}{\frac{\partial T_{2}}{\partial z}|}_{z=z_{2}}=h_{2}T_{2},\\ {\frac{\partial T_{i}}{\partial x}|}_{x=0,\;a}=0,\;i=1,2,\\ {\frac{\partial T_{i}}{\partial y}|}_{y=0,\;a}=0,\;i=1,2. \end{array}\right..$$

Considering the perfect contact at the interface of two layers, the heat continuity condition can be written by

(5) $$\left\{ \begin{array}{l} \;k_{z1}{\frac{\partial T_{1}}{\partial z}|}_{z=z_{1}{}^{-}}=k_{z2}{\frac{\partial T_{2}}{\partial z}|}_{z=z_{1}{}^{+}},\\ {\;T_{1}|}_{z=z_{1}{}^{-}}={T_{2}|}_{z=z_{1}{}^{+}}. \end{array}\right.$$

The initial temperature condition of the structure is set by

(6) $${T_{i}|}_{t=0}=0\;,\;i=1,2.$$

In order to calculate the inhomogeneous equation in Equation (1), the homogeneous equation should be taken into consideration at first, which gives

(7) $$\left\{ \begin{array}{ll} k_{x1}\frac{\partial^{2}T}{\partial x^{2}}+k_{y1}\frac{\partial^{2}T}{\partial y^{2}}+k_{z1}\frac{\partial^{2}T}{\partial z^{2}}=\rho_{1}C_{p1}\frac{\partial T}{\partial t}, & 0<z<z_{1},\;0<x<a,\;0<y<a,\\ k_{x2}\frac{\partial^{2}T}{\partial x^{2}}+k_{y2}\frac{\partial^{2}T}{\partial y^{2}}+k_{z2}\frac{\partial^{2}T}{\partial z^{2}}=\rho_{2}C_{p2}\frac{\partial T}{\partial t}, & z_{1}<z<z_{2},\;0<x<a,\;0<y<a. \end{array}\right..$$

Based on the method of separation of variables, the solution of Equation (1) can be obtained as

(8) $$T_{i}(x,y,z,t)=X_{i}(x)Y_{i}(y)Z_{i}(z)\Phi_{i}(t)\begin{array}{cc} , & i=1,2 \end{array}.$$

Substituting Equation (8) in Equations (4), (5) and (7), the separation equations for the function *X*(*x*) and *Y*(*y*) are

(9) $$\left\{ \begin{array}{l} \frac{1}{X_{i}(x)}\frac{\partial^{2}X_{i}}{\partial x^{2}}=-\omega^{2},\;0<x<a,\\ \frac{1}{Y_{i}(y)}\frac{\partial^{2}Y_{i}}{\partial y^{2}}=-\psi^{2},0<y<a,\\ {\frac{\partial X_{i}}{\partial x}|}_{x=0,\;a}=0,\;{\frac{\partial Y_{i}}{\partial y}|}_{y=0,\;a}=0,\;i=1,2. \end{array}\right.,$$

and the separation equation for *Z*(*z*) are

(10) $$\left\{ \begin{array}{l} \frac{1}{Z_{1}(x)}\frac{\partial^{2}Z_{1}}{\partial z^{2}}=-\zeta_{1}{}^{2}\begin{array}{cc} , & 0<z<z_{1} \end{array},\\ \frac{1}{Z_{2}(x)}\frac{\partial^{2}Z_{2}}{\partial z^{2}}=-\zeta_{2}{}^{2}\begin{array}{cc} , & z_{1}<z<z_{2} \end{array},\\ k_{z1}{\frac{\partial Z_{1}}{\partial z}|}_{z=0}=h_{1}Z_{1},-k_{z2}{\frac{\partial Z_{2}}{\partial z}|}_{z=z_{2}}=h_{2}Z_{2},\\ {Z_{1}|}_{z=z_{1}{}^{-}}={Z_{2}|}_{z=z_{1}{}^{+}},\;k_{z1}{\frac{\partial Z_{1}}{\partial z}|}_{z=z_{1}{}^{-}}=k_{z2}{\frac{\partial Z_{2}}{\partial z}|}_{z=z_{1}{}^{+}}. \end{array}\right..$$

The separation equation for time item is

(11) $$\frac{1}{\Phi_{i}(t)}\frac{\partial \Phi_{i}}{\partial t}=-\lambda^{2}.$$

The coefficient can be obtained by Equation (8) as following

(12) $$\lambda^{2}=\frac{k_{z1}}{\rho_{1}c_{P1}}\left(\frac{k_{x1}}{k_{z1}}\omega^{2}+\frac{k_{y1}}{k_{z1}}\psi^{2}+\zeta_{1}{}^{2}\right)=\frac{k_{z2}}{\rho_{2}c_{p2}}\left(\frac{k_{x2}}{k_{z2}}\omega^{2}+\frac{k_{y2}}{k_{z2}}\psi^{2}+\zeta_{2}{}^{2}\right).$$

Based on the boundary conditions in Equations (9) and (10), the common solutions for *X*, *Y* and *Z* can be expressed as

(13) $$\left\{ \begin{array}{l} X_{1n}=X_{2n}=\cos(\omega_{n}x),\;\;\;\omega_{n}=\frac{n\pi }{a},\\ Y_{1m}=Y_{2m}=\cos(\psi_{m}y),\;\;\;\psi_{m}=\frac{m\pi }{a},\\ Z_{1p}=A\cos(\zeta_{1}z)+B\sin(\zeta_{1}z),\\ Z_{2p}=C\cos(\zeta_{2}z)+D\sin(\zeta_{2}z). \end{array}\right.$$

By substituting $\omega_{n}$ and $\psi_{m}$ in Equation (13) into Equation (12), the eigenvalues $\zeta_{1}$ and $\zeta_{2}$ can written by

(14) $$\left\{ \begin{array}{l} \zeta_{1}=\sqrt{\frac{\rho_{1}c_{p1}}{k_{z1}}\lambda_{nmp}^{2}-\frac{k_{x1}}{k_{z1}}{\left(\frac{n\pi }{a}\right)}^{2}-\frac{k_{y1}}{k_{z1}}{\left(\frac{m\pi }{a}\right)}^{2}},\\ \zeta_{2}=\sqrt{\frac{\rho_{2}c_{p2}}{k_{z2}}\lambda_{nmp}^{2}-\frac{k_{x2}}{k_{z2}}{\left(\frac{n\pi }{a}\right)}^{2}-\frac{k_{y2}}{k_{z2}}{\left(\frac{m\pi }{a}\right)}^{2}}. \end{array}\right.$$

Substituting *Z*_1*p*_ and *Z*_2*p*_ into the separation boundary condition in Equation (10), it can be obtained by:

(15) $$\left[\begin{array}{cccc} -h_{1} & k_{z1}\zeta_{1} & 0 & 0\\ 0 & 0 & k_{z2}\zeta_{2}\sin\left(\zeta_{2}z_{2}\right)-h_{2}\cos\left(\zeta_{2}z_{2}\right) & -k_{z2}\zeta_{2}\cos(\zeta_{2}z_{2})-h_{2}\sin(\zeta_{2}z_{2})\\ \cos(\zeta_{1}z_{1}) & \sin(\zeta_{1}z_{1}) & -\cos(\zeta_{2}z_{1}) & -\sin(\zeta_{2}z_{1})\\ -k_{z1}\zeta_{1}\sin(\zeta_{1}z_{1}) & k_{z1}\zeta_{1}\cos(\zeta_{1}z_{1}) & k_{z2}\zeta_{2}\sin(\zeta_{2}z_{1}) & -k_{z2}\zeta_{2}\cos(\zeta_{2}z_{1}) \end{array}\right]\left[\begin{array}{c} A\\ B\\ C\\ D \end{array}\right]=\left[\begin{array}{c} 0\\ 0\\ 0\\ 0 \end{array}\right]$$

By setting the coefficient *A* = 1, then the *B*, *C* and *D* can be calculated, and *Z*_1*p*_ and *Z*_2*p*_ can be expressed as

(16) $$\left\{ \begin{array}{l} Z_{1p}=\cos(\zeta_{1}z)+\frac{h_{1}}{k_{z1}\zeta_{1}}\sin(\zeta_{1}z),\\ Z_{2p}=\left[\cos(\zeta_{1}z_{1})+\frac{h_{1}}{k_{z1}\zeta_{1}}\sin(\zeta_{1}z_{1})\right]\cos\left[\zeta_{2}(z-z_{1})\right]\\ +\frac{k_{z1}\zeta_{1}}{k_{z2}\zeta_{2}}\left[\frac{h_{1}}{k_{z1}\zeta_{1}}\cos(\zeta_{1}z_{1})-\sin(\zeta_{1}z_{1})\right]\sin\left[\zeta_{2}(z-z_{1})\right]. \end{array}\right.$$

From Equation (15), the eigenvalue equation including $\zeta_{1}$ and $\zeta_{2}$ can be calculated by

(17) $$\frac{k_{z2}\zeta_{2}\tan[\zeta_{2}(z_{2}-z_{1})]-h_{2}}{h_{2}\tan[\zeta_{2}(z_{2}-z_{1})]+k_{z2}\zeta_{2}}+(\frac{\zeta_{1}k_{z1}}{\zeta_{2}k_{z2}})(\frac{k_{z1}\zeta_{1}\tan(\zeta_{1}z_{1})-h_{1}}{h_{1}\tan(\zeta_{1}z_{1})+k_{z1}\zeta_{1}})=0$$

Therefore, the temperature increment satisfying the homogeneous governing equation can be obtained as

(18) $$T_{i}\left(x,y,z,t\right)=\sum_{p=1}^{\infty }\sum_{n=0}^{\infty }\sum_{m=0}^{\infty }X_{n}(\omega_{n}x)Y_{m}(\psi_{m}y)Z_{ip}(z)\Phi (t),\;i=1,\;2.$$

Submitting Equation (18) into Equation (1), the following relation can be obtained:

(19) $$\begin{array}{l} \sum_{p=1}^{\infty }\sum_{n=0}^{\infty }\sum_{m=0}^{\infty }X_{n}(\omega_{n}x)Y_{m}(\psi_{m}y)Z_{1p}(z)\left(\frac{\partial \Phi (t)}{\partial t}+\lambda^{2}\Phi (t)\right)=0\\ \sum_{p=1}^{\infty }\sum_{n=0}^{\infty }\sum_{m=0}^{\infty }X_{n}(\omega_{n}x)Y_{m}(\psi_{m}y)Z_{2p}(z)\left(\frac{\partial \Phi (t)}{\partial t}+\lambda^{2}\Phi (t)\right)=\frac{1}{\rho_{2}c_{p2}}Q \end{array}$$

Based on the orthogonality of eigenfunctions, the operators *S*_1_ and *S*_2_ can be expressed as

(20) $$\left\{ \begin{array}{l} S_{1}=\rho_{1}c_{p1}\int_{0}^{a}\int_{0}^{a}\int_{0}^{z_{1}}X_{n}(\omega_{n}x)Y_{m}(\psi_{m}y)Z_{1p}(z)dxdydz\\ S_{2}=\rho_{2}c_{p2}\int_{0}^{a}\int_{0}^{a}\int_{z_{1}}^{z_{2}}X_{n}(\omega_{n}x)Y_{m}(\psi_{m}y)Z_{2p}(z)dxdydz \end{array}\right.$$

Therefore, the equation about Φ(t) can be written by

(21) $$\left(\frac{\partial \Phi (t)}{\partial t}+\lambda^{2}\Phi (t)\right)=\frac{F_{nm}H_{p}}{N_{n}(\omega_{n})N_{m}(\psi_{m})N_{p}}Q_{0}\begin{array}{cc} , & i=1,2 \end{array},$$

where

(22) $$\begin{array}{l} F_{nm}=\int_{0}^{a}\int_{0}^{a}X_{n}(\omega_{n}x)Y_{m}(\psi_{m}y)Q_{1}(x,y)dxdy\\ =\int_{0}^{W_{1}}X_{n}(\omega_{n}x)dx\int_{a-W_{2}-\Delta t}^{a-W_{2}}Y_{m}(\psi_{m}y)dy\\ +\int_{W_{1}}^{W_{1}+\Delta t}X_{n}(\omega_{n}x)dx\int_{W_{2}}^{a-W_{2}}Y_{m}(\psi_{m}y)dy\\ +\int_{W_{1}+\Delta t}^{a}X_{n}(\omega_{n}x)dx\int_{W_{2}}^{W_{2}+\Delta t}Y_{m}(\psi_{m}y)dy \end{array}$$

(23) $$H_{p}=\int_{z_{1}}^{z_{2}}Z_{2p}(z)Q_{2}(z)dz=Z_{2p}(z_{1})$$

(24) $$N_{n}(\omega_{n})=\int_{0}^{a}X_{n}^{2}(\omega_{n}x)dx=\{\begin{array}{c} a\begin{array}{cc} , & \omega_{n}=0 \end{array}\\ a/2\begin{array}{cc} , & \omega_{n}\neq 0 \end{array} \end{array}$$

(25) $$N_{m}(\psi_{m})=\int_{0}^{a}Y_{m}^{2}(\psi_{m}y)dx=\{\begin{array}{c} a\begin{array}{cc} , & \psi_{m}=0 \end{array}\\ a/2\begin{array}{cc} , & \psi_{m}\neq 0 \end{array} \end{array}$$

(26) $$N_{p}=\rho_{1}c_{p1}\int_{0}^{z_{1}}Z_{1p}^{2}(z)dz+\rho_{2}c_{p2}\int_{z_{1}}^{z_{2}}Z_{2p}^{2}(z)dz$$

According to the initial condition in Equation (6), Φ(t) can be calculated by

(27) $$\Phi \left(t\right)=\frac{F_{nm}H_{p}}{N_{n}(\omega_{n})N_{m}(\psi_{m})N_{p}}Q_{0}P_{nmp},$$

where

(28) $$P_{nmp}=\int_{\tau =0}^{t}e^{-\lambda_{nmp}^{2}(t-\tau )}Q_{3}(\tau )d\tau =\frac{1-e^{-\lambda_{nmp}^{2}t}}{\lambda_{nmp}^{2}}.$$

Therefore, the temperature increment of the stretchable rectangular heater can by expressed as

(29) $$T_{i}\left(x,y,z,t\right)=\sum_{p=1}^{\infty }\sum_{n=0}^{\infty }\sum_{m=0}^{\infty }Q_{0}\frac{X_{n}(\omega_{n}x)Y_{m}(\psi_{m}y)Z_{ip}(z)}{N_{n}(\omega_{n})N_{m}(\psi_{m})N_{p}}F_{nm}H_{p}P_{nmp}\begin{array}{cc} , & i=1,2 \end{array}.$$

## 3. Results and Discussion

The analytical model of transient heat conduction based on orthotropic characteristics is verified by FEA in this section. The stretchable rectangular copper heater is encapsulated in the middle layer by the encapsulation and the substrate, which are composed of 0.5 mm thick silicone (e.g., Ecoflex00-10, Smooth-On, Inc., Macungie, PA, USA). The width and thickness of the heater are 0.2 mm and 1 nm, respectively, as shown in Figure 2c. The dimensions of structural parameters *a*, *W*_1_ and *W*_2_ marked in the figure are 3 mm, 1.4 mm and 0.8 mm, respectively. The input power applied to the heater of one quarter period is 0.02 W, and the surface heat flux is 2.381 × 10^4^ W·m^−2^. The natural convective heat transfer coefficient above the encapsulation layer and below the substrate is taken as 15 W·m^−2^·K^−1^. The conductivity, density, and specific heat capacity of Ecoflex and copper used in this model are 0.16 W·m^−1^·K^−1^, 1.07 × 10^3^ kg·m^−3^, 1.7 × 10^3^ J·kg^−1^·K^−1^ and 394 W·m^−1^·K^−1^, 8.92 × 10^3^ kg·m^−3^, 0.39 × 10^3^ J·kg^−1^·K^−1^, respectively. A 3D transient heat conduction FEA model based on ABAQUS is established. The heater is discretized using a shell element DS4 with a defined thickness of 1 nm. The encapsulation and substrate are discretized using DC3C8 element. The element dimension of the whole model was set to a consistent 0.05 mm with a total number of 10,000, and the convergence of the simulation results was verified. Compared with the much smaller unit size of 0.005 mm, the deviation of the simulation results was less than 0.1%.

**Figure 3:**
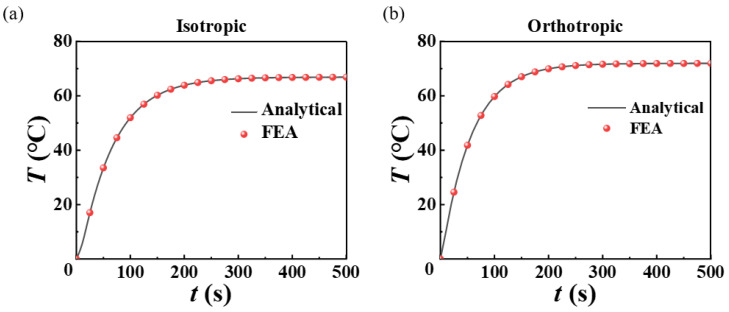
Evolution of temperature increment with time at the substrate geometric center of (**a**) isotropic and (**b**) orthotropic heat conduction system with serpentine heater.

The transient heat conduction characteristics of stretchable heater systems and the homogenization effect of orthotropic characteristics on temperature distribution are investigated via above analytical and FEA models. Since the temperature distribution at the interface between the heater and the skin is a main indicator for the hyperthermia effect, we focused on the temperature results at the substrate bottom surface. For the substrate bottom surface of the periodic stretchable heater, the temperature time evolution of the isotropic and orthotropic heat conduction of the repeatable unit geometric center (point marked in red in Figure 2b) are demonstrated in Figure 3a,b, respectively. For substrates with isotropic heat conduction characteristics, the thermal conductivity in three directions is the same 0.16 W·m^−1^·K^−1^. For the orthotropic substrate, the thermal conductivity in the *y*-direction is increased to 1.6 W·m^−1^·K^−1^, while the thermal conductivity in other two directions remains unchanged. Here, the time when the temperature reaches 99% of the steady-state temperature is defined as the steady-state time. The results of temperature evolution with time shown in Figure 3 show that the steady-state temperature and steady-state time of the geometric center at the bottom surface of the isotropic substrate are 66.9 °C and 292 s, respectively. The steady-state temperature of the orthotropic substrate is 71.9 °C, and the steady-state time is 256 s. The analytical model results and the FEA results are verified by mutual matching.

**Figure 4:**
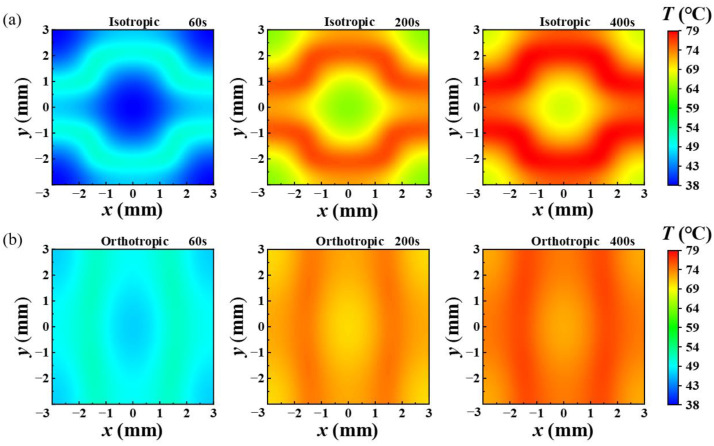
Transient temperature variation of (**a**) isotropic and (**b**) orthotropic substrates bottom surface (t: 60 s, 200 s and 400 s).

To show the effect of orthotropic substrates in improving the uniformity of temperature distribution, Figure 4 demonstrates the transient temperature distributions variation of isotropic and orthotropic substrates bottom surface (t: 60 s, 200 s and 400 s). As can be seen from the temperature distribution of the orthotropic heat conduction substrate in Figure 4a, the temperature distribution at different times has obvious inhomogeneity with the temperature difference of over 10 °C. For the orthotropic substrate shown in Figure 4b, under the same input power condition, the temperature distribution uniformity is significantly improved, and the temperature difference seems to be no more than 3 °C.

**Figure 5:**
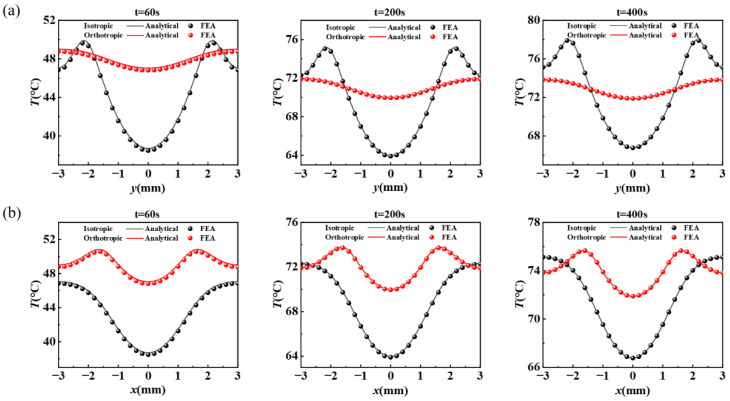
Temperature distributions along the (**a**) *y*-direction and (**b**) *x*-direction of the isotropic and orthotropic substrate bottom surface at different times (t: 60 s, 200 s and 400 s).

In order to quantitatively investigate the effect of orthotropic substrate on improving the uniformity of temperature distribution of stretchable heaters, Figure 5 demonstrates the temperature distributions along the *y*-direction (*x* = 0 mm) and *x*-direction (*y* = 0 mm) of the isotropic and orthotropic substrate bottom surface at different times (t: 60 s, 200 s and 400 s). For the temperature distribution along the *y*-direction (*x* = 0 mm) path, the heat conduction system of isotropic substrate at different times has the highest temperature at the heater pattern metal and the lowest temperature at the geometric center of the unit. In 60 s, the temperature varies from 49.9 °C to 38.7 °C, and the temperature difference is 11.2 °C. In 200 s, the temperature varies from 75.2 °C to 64.0 °C, and the temperature difference is 11.8 °C. In 400 s, the temperature varies from 78.0 °C to 66.8 °C, and the temperature difference is 11.2 °C. For the orthotropic heat conduction system, the temperature distribution and the difference along the *y*-direction (*x* = 0 mm) path varies significantly, and the highest temperature point shifts to the edge of the unit. In 60 s, the temperature varies from 49.0 °C to 47.0 °C, and the temperature difference is 2.0 °C. In 200 s, the temperature varies from 72.0 °C to 70.0 °C, and the temperature difference is 2.0 °C. In 400 s, the temperature varies from 73.8 °C to 71.9 °C, and the temperature difference is 1.9 °C. In the three selected times, the temperature difference decreased from more than 11 °C to 2 °C and below, with a reduction ratio of more than 82%. The results indicate that the orthotropic heat conduction strategy of increasing the thermal conductivity in the *y*-direction has a markable effect on the homogenization of the temperature distribution in the *y*-direction of the stretchable heater. The temperature distribution uniformity along the *x*-direction (*y* = 0 mm) path of the substrate with different heat conduction strategies also shows obvious differences. For the isotropic substrate, the temperature difference at three times is about 8.3 °C (60 s: 47.0 °C to 38.7 °C; 200 s: 72.3 °C to 64.0 °C; 400 s: 75.1 °C to 66.8 °C). For the orthotropic substrate, the difference drops to about 3.8 °C (60 s: 50.8 °C to 47.0 °C; 200 s: 73.8 °C to 70.0 °C; 400 s: 75.7 °C to 71.9 °C), with a reduction ratio of around 54%. This indicates that increasing the thermal conductivity in the *y*-direction can improve the temperature distribution uniformity in the *x*-direction, but the homogenization effect is inferior to that in the *y*-direction.

**Figure 6:**
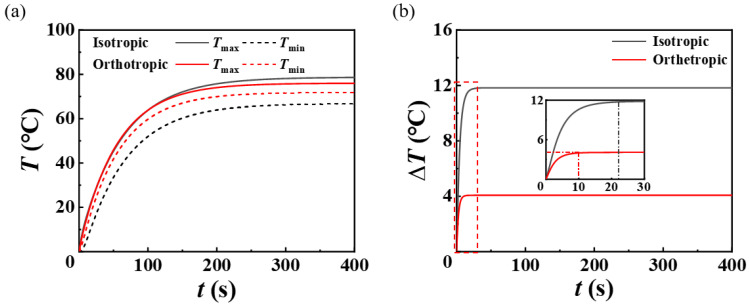
Evolution of (**a**) the maximum and minimum temperatures and (**b**) the temperature difference with time at the bottom isotropic and orthotropic substrate surface.

Since the temperature difference is the main index we are most concerned about, we extract the evolution of the maximum temperature and the minimum temperature at the bottom isotropic and orthotropic substrate surface with time, and then obtain the evolution of the temperature difference with time, as shown in Figure 6. The results demonstrated in Figure 6a show that the orthotropic heat conduction strategy can reduce the maximum temperature and increase the minimum temperature of the substrate surface, thereby achieving the purpose of reducing the temperature difference and homogenizing the temperature distribution. The temperature difference decreases from 11.8 °C to 4.0 °C, and the time for the temperature difference to reach stability is shortened from 22 s for isotropic conduction to 10 s for orthotropic conduction, as shown in Figure 6b. This indicates that the orthotropic heat conduction of the stretchable heater is a beneficial strategy for improving the temperature uniformity and accelerating the heat transfer system to reach the steady state.

**Figure 7:**
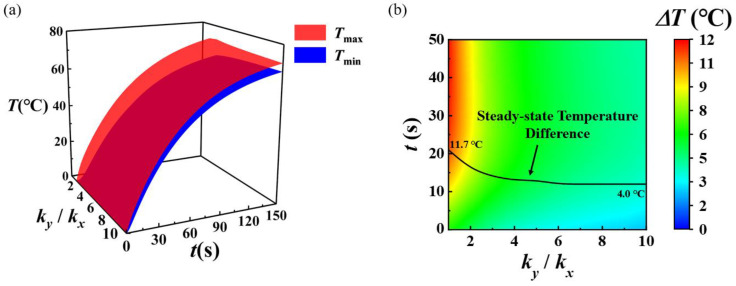
Evolution of (**a**) the maximum and minimum temperatures and (**b**) the temperature difference with time and the ratio of *k_y_/k_x_* at the substrate bottom surface.

As it is concluded in Figure 5, increasing the thermal conductivity in the *y*-direction does not demonstrate the same effect of temperature homogenization in the *x*-direction and the *y*-direction. In order to systematically explore the influence of thermal conductivity in *x* and *y* directions on temperature distribution, the evolution of temperature with time and thermal conductivity ratio *k_y_/k_x_* is calculated, as shown in Figure 7, including the temperature extremum evolution and the corresponding temperature difference. According to the conclusion obtained in Figure 3 and Figure 6, the time for the transient heat transfer system to reach the steady state exceeds 200 s, while the time for the temperature difference to reach the stable state is much shorter. The steady-state temperature difference value with different thermal conductivity ratios is marked in Figure 7b. With the increment of thermal conductivity ratio *k_y_/k_x_* from 1 to 10, the time for the temperature difference to reach the steady state is reduced from 22 s to 12 s (*k_y_/k_x_*: 1 to 6), and then remains basically stable (*k_y_/k_x_*: 6 to 10). The steady-state temperature difference decreases continuously with the increase in the thermal conductivity ratio, from 11.7 °C to 4.0 °C.

**Figure 8:**
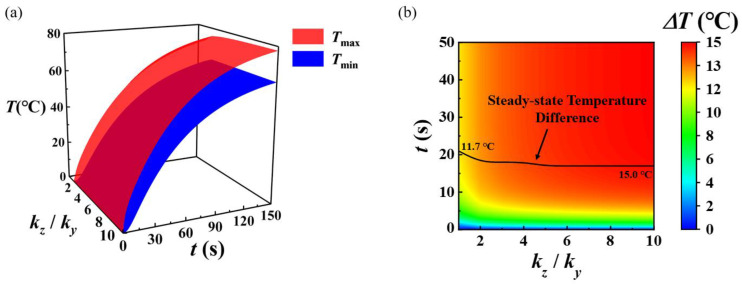
Evolution of (**a**) the maximum and minimum temperatures and (**b**) the temperature difference with time and the ratio of *k_z_/k_y_* at the substrate bottom surface.

Further, the evolution of temperature with time and thermal conductivity ratio *k_z_/k_y_* is plotted in Figure 8 to explore the influence of off-plane (i.e., *z*-direction) thermal conductivity on enhancing the uniformity of temperature distribution. With the increment of thermal conductivity ratio *k_z_/k_y_* from 1 to 10, the time for the temperature difference to reach the steady state is slightly reduced from 22 s to 17 s. The steady-state temperature difference increases from 11.7 °C to 15.0 °C with the increase in the thermal conductivity ratio, which indicates that the temperature distribution uniformity is further deteriorated. The reason for this phenomenon is that the in-plane thermal conductivity of the substrate remains unchanged, the off-plane thermal conductivity is increased, and the heat flow is regulated to the off-plane direction, resulting in the temperature distribution on the bottom substrate surface much more easily influenced by the patterned configuration of the stretchable heater.

## 4. Conclusions

This paper proposes an analytical model for transient heat conduction of stretchable rectangular heaters based on orthotropic transfer characteristics, which is validated by FEA. The orthotropic heat transfer characteristics are utilized to enhance the temperature inhomogeneity of inorganic stretchable heaters, which is caused by the space of the patterned stretchable electrodes. The temperature distribution at the interface with the skin is the key to the effect of the hyperthermia electrode. This paper focuses on the temperature distribution at the bottom substrate surface of the stretchable heater and its evolution with time. The results show that increasing the thermal conductivity in the *y*-direction can significantly improve the uniformity of temperature distribution during the heating process, and the uniformity effect in the *y*-direction is better than that in the *x*-direction. This orthotropic heat transfer strategy can significantly shorten the time for temperature difference to reach the steady state. Furthermore, the influence of thermal conductivity ratios in different directions (*k_y_/k_x_* and *k_z_/k_y_*) on temperature distribution and time evolution is investigated. Increasing the thermal conductivity in the in-plane direction is helpful to improve the uniformity of temperature distribution, while increasing the thermal conductivity in the off-plane direction has the contrary effect. The establishment of a transient heat transfer model in this paper and the investigation of temperature homogenization effect based on it have valuable guiding significance for the structure design of stretchable heaters and the mechanism research of transient heat transfer process.

## Data Availability

The data presented in this study are available on reasonable request from the corresponding author.
